# Crystal structure of 4-[(*E*)-(4-nitro­benzyl­idene)amino]­phenol

**DOI:** 10.1107/S2056989015000511

**Published:** 2015-01-17

**Authors:** Zeliha Atioğlu, Mehmet Akkurt, Aliasghar Jarrahpour, Edris Ebrahimi, Orhan Büyükgüngör

**Affiliations:** a İlke Education and Health Foundation, Cappadocia Vocational College, The Medical Imaging Techniques Program, 50420 Mustafapaşa, Ürgüp, Nevşehir, Turkey; b Department of Physics, Faculty of Sciences, Erciyes University, 38039 Kayseri, Turkey; c Department of Chemistry, College of Sciences, Shiraz University, 71454 Shiraz, Iran; d Department of Physics, Faculty of Arts and Sciences, Ondokuz Mayıs University, 55139 Samsun, Turkey

**Keywords:** crystal structure, whole-mol­ecule disorder, nitro­aromatic compounds, hydrogen bonding, C—H⋯π inter­actions, π–π stacking inter­actions

## Abstract

The asymmetric unit of the title compound, C_13_H_10_N_2_O_3_, contains four independent mol­ecules (I, II, III and IV). Mol­ecule IV shows whole-mol­ecule disorder over two sets of adjacent sites in a 0.669 (10):0.331 (10) ratio. The dihedral angles between the aromatic rings are 32.30 (13)° in mol­ecule I, 2.24 (14)° in II, 41.61 (13)° in III, 5.0 (5)° in IV (major component) and 10.2 (3)° in IV (minor component). In the crystal, mol­ecules are linked into layers lying parallel to (024) by C—H⋯O and O—H⋯O inter­actions. The layers inter­act by C—H⋯π and weak aromatic π–π stacking inter­actions [centroid–centroid distances = 3.8476 (16), 3.725 (3) and 3.733 (5) Å].

## Related literature   

For background to the importance of Schiff bases and nitro­aromatic compounds and their uses, see: Docampo (1990 ▸); Ma *et al.* (2003 ▸); Purohit & Basu (2000 ▸); Safwat *et al.* (1988 ▸); Tarafder *et al.* (2002 ▸). For related structures, see: Valkonen *et al.* (2012 ▸); Akkurt *et al.* (2013 ▸, 2014 ▸); Atioğlu *et al.* (2014 ▸).
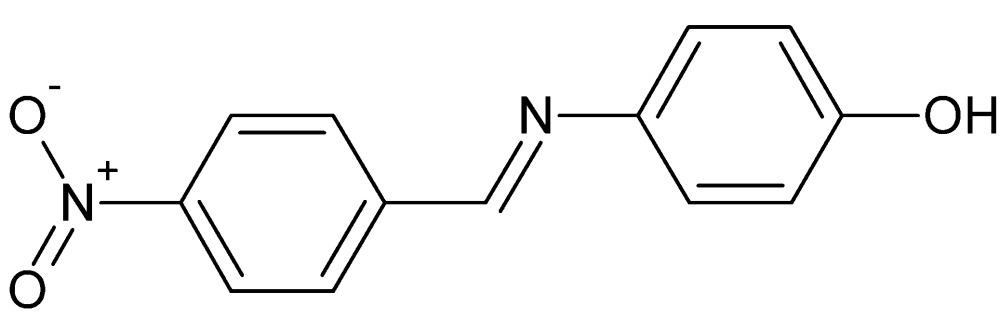


## Experimental   

### Crystal data  

C_13_H_10_N_2_O_3_
*M* *_r_* = 242.23
Triclinic, 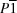

*a* = 12.3449 (6) Å
*b* = 13.4266 (6) Å
*c* = 15.7404 (7) Å
α = 72.926 (4)°
β = 67.390 (4)°
γ = 76.824 (4)°
*V* = 2282.7 (2) Å^3^
*Z* = 8
Mo *K*α radiation
μ = 0.10 mm^−1^
*T* = 296 K
0.53 × 0.49 × 0.41 mm

### Data collection  

Stoe IPDS 2 diffractometer
Absorption correction: integration (*X-RED32*; Stoe & Cie, 2002 ▸) *T*_min_ = 0.952, *T*_max_ = 0.974
33476 measured reflections
10470 independent reflections
5234 reflections with *I* > 2σ(*I*)
*R*_int_ = 0.099

### Refinement  

*R*[*F* ^2^ > 2σ(*F* ^2^)] = 0.070
*wR*(*F* ^2^) = 0.197
*S* = 0.96
10470 reflections
680 parameters
5 restraints
H-atom parameters constrained
Δρ_max_ = 0.51 e Å^−3^
Δρ_min_ = −0.26 e Å^−3^

### 

Data collection: *X-AREA* (Stoe & Cie, 2002 ▸); cell refinement: *X-AREA*; data reduction: *X-RED32* (Stoe & Cie, 2002 ▸); program(s) used to solve structure: *SUPERFLIP* (Palatinus & Chapuis, 2007 ▸); program(s) used to refine structure: *SHELXL97* (Sheldrick, 2008 ▸, 2015 ▸); molecular graphics: *ORTEP-3 for Windows* (Farrugia, 2012 ▸); software used to prepare material for publication: *WinGX* (Farrugia, 2012 ▸).

## Supplementary Material

Crystal structure: contains datablock(s) global, I. DOI: 10.1107/S2056989015000511/hb7349sup1.cif

Structure factors: contains datablock(s) I. DOI: 10.1107/S2056989015000511/hb7349Isup2.hkl

Supporting information file. DOI: 10.1107/S2056989015000511/hb7349Isup3.cml

**Fig. 1.**
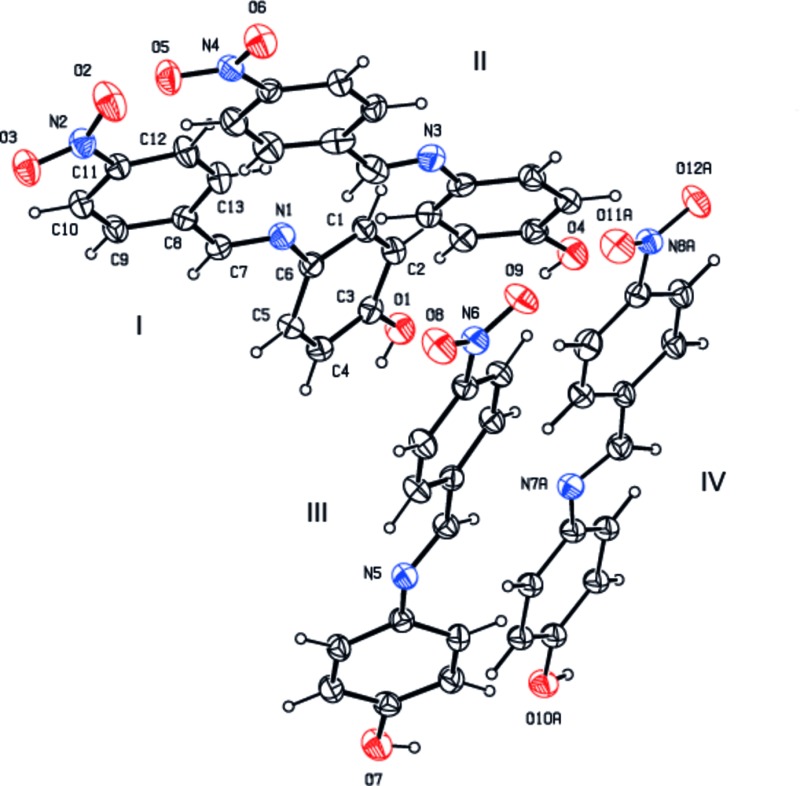
The molecular structure of the title compound (I) with displacement ellipsoids drawn at the 20% probability level. Only the major component of the disorder is shown.

**Fig. 2.**
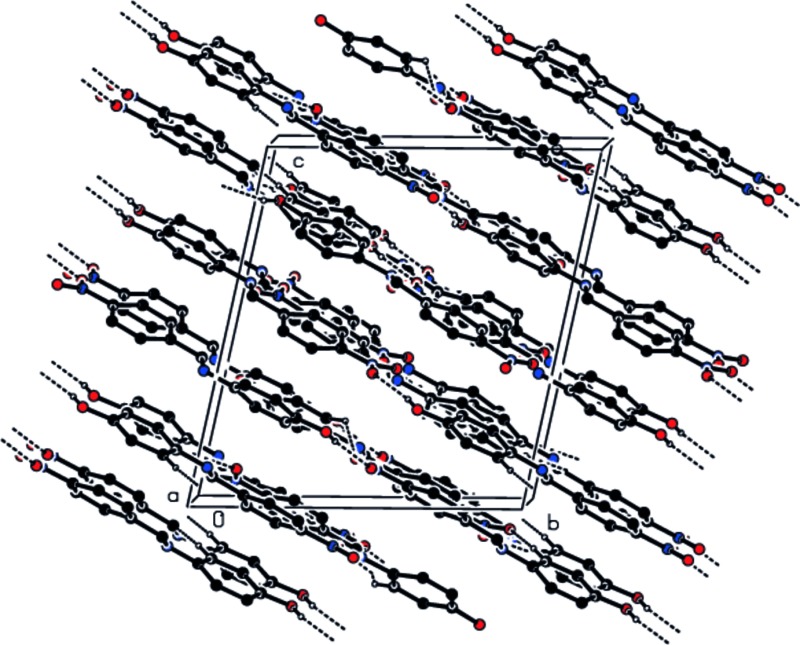
View of the hydrogen bonding and molecular packing of (I) along a axis. Only H atoms involved in H bonding and atoms of the major disorder component are shown.

. DOI: 10.1107/S2056989015000511/hb7349fig1.tif
The mol­ecular structure of the title compound (I) with displacement ellipsoids drawn at the 20% probability level. Only the major component of the disorder is shown.

a . DOI: 10.1107/S2056989015000511/hb7349fig2.tif
View of the hydrogen bonding and mol­ecular packing of (I) along *a* axis. Only H atoms involved in H bonding and atoms of the major disorder component are shown.

CCDC reference: 1042888

Additional supporting information:  crystallographic information; 3D view; checkCIF report

## Figures and Tables

**Table 1:** Hydrogen-bond geometry (, ) *Cg*2, *Cg*3, *Cg*4 and *Cg*8 are the centroids of the C8C13, C14C19, C21C26 and C47*A*C52*A* benzene rings, respectively.

*D*H*A*	*D*H	H*A*	*D* *A*	*D*H*A*
O1H*O*1O9^i^	0.81	2.05	2.836(3)	163
O4H*O*4O12*A* ^i^	0.82	2.16	2.89(3)	148
O7H*O*7O3^ii^	0.82	2.06	2.832(4)	158
C1H1O6^iii^	0.93	2.53	3.417(4)	159
C14H14O2^iii^	0.93	2.59	3.289(4)	132
C2H2*Cg*2^iv^	0.93	3.00	3.610(3)	125
C15H15*Cg*8^v^	0.93	2.99	3.665(4)	131
C36H36*Cg*3	0.93	2.81	3.487(3)	130
C43*A*H43*A* *Cg*4^vi^	0.93	2.80	3.539(5)	137
C43H43*Cg*4^vi^	0.93	2.94	3.590(11)	128

Symmetry codes: (i) 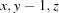; (ii) 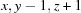; (iii) 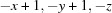; (iv) 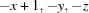; (v) 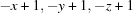; (vi) 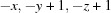.

## supplementary crystallographic information

### S1. Comment

Schiff bases are potentially biologically active compounds and have been reported to possess antifungal, anticancer, anticonvulsant and diuretic activities (Safwat *et al.*, 1988). Schiff bases derived from various heterocycles were reported to possess cytotoxic activity (Tarafder *et al.*, 2002). It is believed that the presence of a nitro group in the *p*-position appears to be an important condition for the development of bacteriostatic activity (Ma *et al.*, 2003). Nitroaromatic compounds are widely used in medicine, industry and agriculture (Purohit *et al.*, 2000). The anti-malarial activity of nitroaromatic compounds was attributed to the formation of reactive oxygen species during flavoenzyme catalyzed redox cycling reactions and/or oxyhemoglobin oxidation (Docampo, 1990).

The crystal structures of molecules I, II and III of the title compound are ordered, but in (IV) a "*whole-disorder molecule*" exists (Fig. 1), with the occupancy of the major component being 0.669 (10).

The hydroxy benzene ring is inclined at an angle of 32.30 (13)° in (I), 2.24 (14)° in (II), 41.61 (13)° in (III), 5.0 (5)° in (IV) and 10.2 (3)° in (IVA) with respect to the nitro benzene ring. In the disordered molecules IV, the dihedral angles between the hydroxy benzene rings and and the nitro benzene rings of the two disorders are 2.4 (4) and 3.6 (4) °, respectively.

In the asymmetric unit, the bond lengths and bond angles in all molecules are similar and comparable with those of the related structures reported by Valkonen *et al.*, 2012; Akkurt *et al.*, 2014; Atioğlu *et al.*, 2014; Akkurt *et al.*, 2013.

In the crystal structure, intermolecular C—H···O and O—H···O hydrogen bonding interactions link the molecules into a two-dimensional layered structure parallel to the (024) plane (Table 1, Fig. 2). C—H···π interactions and π–π stacking interactions [*Cg*3···*Cg*4 (1 - *x*, 1 - *y*, -*z*) = 3.8476 (16) Å, *Cg*6···*Cg*7 (*x*, *y*, *z*) = 3.725 (3) Å and *Cg*6···*Cg*9 (*x*, *y*, *z*) = 3.733 (5) Å; *Cg*3, *Cg*4, *Cg*6, *Cg*7 and *Cg*9 are the centroids of the (C14–C19), (C21–C26), (C34–C39), (C40A–C45A) and (C40–C45) benzene rings, respectively] between the layers stabilize the molecular packing.

### S2. Experimental

*p*-Nitrobenzaldehyde (3.0 mmol, 0.46 g) was added to 4-aminophenol (3.0 mmol, 0.33 g) in EtOH (10 ml) refluxed for 4 h. After filtration and recrystallization from EtOH the title Schiff base gave colourless crystals in 85% yield. Mp: 449–451 K. IR (KBr, cm^-1^): 3448 (OH), 1627 (C=N). ^1^H-NMR (250 MHz, CDCl_3_), δ (p.p.m.): 6.83 (ArH, 2H, d, J=1.6 Hz), 7.30 (ArH, 2H, d, J=8.8 Hz), 8.13 (ArH, 2H, d, J=8.8 Hz), 8.33 (ArH, 2H, d, J=8.3 Hz), and 8.80 (CH=N,1*H*, s), 9.66 (OH, s, 1H). ^13^C-NMR (62.9 MHz, DMSO), δ (p.p.m.):116.5, 123.8, 124.6, 129.7, 142.3, 142.8, 149.04, 155.3 (aromatic carbons),157.9 (C=N).

### S3. Refinement

The molecule IV in the asymmetric unit is "*whole-molecule disorder*" over two sets of sites with occupancies in a ratio of 0.669 (10):0.331 (10). All H atoms bound to C atoms and the hydroxyl H atoms (H10A and H1A0) in the disordered molecule IV were positioned geometrically. The H atoms of the hydroxyl groups in the other molecules were located from electron-density maps, but they were calculated at their idealized positions and all were refined using a riding model with C—H = 0.93 Å, O—H = 0.82 Å and *U*_iso_(H) = 1.2*U*_eq_(C) and 1.5*U*_eq_(O)

### Crystal data

**Table:**

C_13_H_10_N_2_O_3_	*Z* = 8
*M**_r_* = 242.23	*F*(000) = 1008
Triclinic, *P*1	*D*_x_ = 1.410 Mg m^−^^3^
Hall symbol: -P 1	Mo *K*α radiation, λ = 0.71073 Å
*a* = 12.3449 (6) Å	Cell parameters from 25151 reflections
*b* = 13.4266 (6) Å	θ = 1.8–27.9°
*c* = 15.7404 (7) Å	µ = 0.10 mm^−^^1^
α = 72.926 (4)°	*T* = 296 K
β = 67.390 (4)°	Prism, colourless
γ = 76.824 (4)°	0.53 × 0.49 × 0.41 mm
*V* = 2282.7 (2) Å^3^	

### Data collection

**Table:**

Stoe IPDS 2 diffractometer	10470 independent reflections
Radiation source: sealed X-ray tube, 12 x 0.4 mm long-fine focus	5234 reflections with *I* > 2σ(*I*)
Plane graphite monochromator	*R*_int_ = 0.099
Detector resolution: 6.67 pixels mm^-1^	θ_max_ = 27.6°, θ_min_ = 1.8°
ω scans	*h* = −16→15
Absorption correction: integration (*X-RED32*; Stoe & Cie, 2002)	*k* = −17→17
*T*_min_ = 0.952, *T*_max_ = 0.974	*l* = −20→20
33476 measured reflections	

### Refinement

**Table:**

Refinement on *F*^2^	5 restraints
Least-squares matrix: full	Hydrogen site location: mixed
*R*[*F*^2^ > 2σ(*F*^2^)] = 0.070	H-atom parameters constrained
*wR*(*F*^2^) = 0.197	*w* = 1/[σ^2^(*F*_o_^2^) + (0.0989*P*)^2^] where *P* = (*F*_o_^2^ + 2*F*_c_^2^)/3
*S* = 0.96	(Δ/σ)_max_ < 0.001
10470 reflections	Δρ_max_ = 0.51 e Å^−^^3^
680 parameters	Δρ_min_ = −0.26 e Å^−^^3^

### Special details

**Table:**

Geometry. Bond distances, angles *etc*. have been calculated using the rounded fractional coordinates. All su's are estimated from the variances of the (full) variance-covariance matrix. The cell e.s.d.'s are taken into account in the estimation of distances, angles and torsion angles
Refinement. Refinement on *F*^2^ for ALL reflections except those flagged by the user for potential systematic errors. Weighted *R*-factors *wR* and all goodnesses of fit *S* are based on *F*^2^, conventional *R*-factors *R* are based on *F*, with *F* set to zero for negative *F*^2^. The observed criterion of *F*^2^ > σ(*F*^2^) is used only for calculating -*R*-factor-obs *etc*. and is not relevant to the choice of reflections for refinement. *R*-factors based on *F*^2^ are statistically about twice as large as those based on *F*, and *R*-factors based on ALL data will be even larger.

### Fractional atomic coordinates and isotropic or equivalent isotropic displacement parameters (Å^2^)

**Table:**

	*x*	*y*	*z*	*U*_iso_*/*U*_eq_	Occ. (<1)
O1	0.50039 (19)	−0.36460 (16)	0.28944 (16)	0.0835 (8)	
O2	0.1795 (2)	0.53357 (19)	−0.1317 (2)	0.1147 (13)	
O3	0.0208 (2)	0.47714 (17)	−0.10965 (19)	0.0933 (10)	
N1	0.3541 (2)	0.04251 (18)	0.12063 (16)	0.0650 (8)	
N2	0.1187 (2)	0.4635 (2)	−0.10289 (18)	0.0718 (9)	
C1	0.5013 (2)	−0.0934 (2)	0.15946 (19)	0.0620 (9)	
C2	0.5401 (2)	−0.1958 (2)	0.1993 (2)	0.0673 (10)	
C3	0.4587 (2)	−0.2649 (2)	0.25257 (19)	0.0615 (9)	
C4	0.3386 (2)	−0.2317 (2)	0.26808 (19)	0.0645 (9)	
C5	0.3021 (2)	−0.1315 (2)	0.22690 (19)	0.0627 (9)	
C6	0.3833 (2)	−0.0606 (2)	0.16962 (18)	0.0587 (9)	
C7	0.2659 (2)	0.0614 (2)	0.09237 (18)	0.0603 (9)	
C8	0.2321 (2)	0.1657 (2)	0.04025 (18)	0.0580 (9)	
C9	0.1387 (2)	0.1805 (2)	0.0077 (2)	0.0640 (10)	
C10	0.1018 (2)	0.2770 (2)	−0.0398 (2)	0.0652 (10)	
C11	0.1614 (2)	0.3603 (2)	−0.05649 (19)	0.0581 (9)	
C12	0.2590 (2)	0.3470 (2)	−0.0295 (2)	0.0708 (10)	
C13	0.2926 (3)	0.2508 (2)	0.0190 (2)	0.0698 (11)	
O4	0.62408 (19)	0.06613 (17)	0.33983 (17)	0.0884 (9)	
O5	0.1439 (2)	0.88143 (19)	−0.10061 (18)	0.0923 (9)	
O6	0.2621 (2)	0.95766 (17)	−0.07833 (19)	0.0932 (9)	
N3	0.4627 (2)	0.4603 (2)	0.15030 (18)	0.0831 (10)	
N4	0.22268 (19)	0.87913 (19)	−0.07013 (16)	0.0643 (8)	
C14	0.5837 (2)	0.3448 (2)	0.2357 (2)	0.0644 (9)	
C15	0.6250 (2)	0.2482 (2)	0.2825 (2)	0.0655 (10)	
C16	0.5807 (2)	0.1583 (2)	0.29194 (19)	0.0639 (9)	
C17	0.4949 (2)	0.1654 (2)	0.2526 (2)	0.0681 (10)	
C18	0.4553 (2)	0.2618 (2)	0.20622 (19)	0.0667 (10)	
C19	0.4968 (2)	0.3534 (2)	0.19782 (18)	0.0628 (9)	
C20	0.3792 (3)	0.4787 (3)	0.1257 (3)	0.0897 (14)	
C21	0.3415 (3)	0.5865 (2)	0.0741 (2)	0.0749 (11)	
C22	0.3926 (2)	0.6749 (3)	0.0635 (2)	0.0764 (13)	
C23	0.3554 (2)	0.7735 (2)	0.01514 (19)	0.0649 (10)	
C24	0.2691 (2)	0.7782 (2)	−0.02215 (18)	0.0562 (8)	
C25	0.2217 (2)	0.6907 (2)	−0.0134 (2)	0.0692 (11)	
C26	0.2566 (3)	0.5968 (3)	0.0359 (2)	0.0811 (12)	
O7	−0.10722 (19)	−0.36719 (16)	0.78633 (17)	0.0856 (9)	
O8	0.1778 (2)	0.53834 (18)	0.3923 (2)	0.1027 (10)	
O9	0.35460 (18)	0.47295 (17)	0.38559 (18)	0.0875 (9)	
N5	0.00532 (19)	0.04052 (17)	0.62254 (16)	0.0615 (8)	
N6	0.2508 (2)	0.46369 (18)	0.40531 (17)	0.0678 (9)	
C27	0.0410 (2)	−0.1359 (2)	0.72341 (19)	0.0608 (9)	
C28	0.0131 (2)	−0.2363 (2)	0.7640 (2)	0.0670 (10)	
C29	−0.0768 (2)	−0.2677 (2)	0.74937 (19)	0.0616 (9)	
C30	−0.1403 (2)	−0.1970 (2)	0.6974 (2)	0.0662 (10)	
C31	−0.1132 (2)	−0.0948 (2)	0.65762 (19)	0.0595 (9)	
C32	−0.0199 (2)	−0.0636 (2)	0.66785 (18)	0.0560 (8)	
C33	0.1125 (2)	0.0584 (2)	0.58926 (19)	0.0595 (9)	
C34	0.1457 (2)	0.1640 (2)	0.54077 (18)	0.0553 (8)	
C35	0.2643 (2)	0.1772 (2)	0.49720 (19)	0.0605 (9)	
C36	0.3001 (2)	0.2740 (2)	0.45158 (19)	0.0593 (9)	
C37	0.2141 (2)	0.3600 (2)	0.44933 (19)	0.0573 (9)	
C38	0.0943 (2)	0.3494 (2)	0.4904 (2)	0.0683 (10)	
C39	0.0611 (2)	0.2517 (2)	0.5364 (2)	0.0649 (10)	
O10A	−0.013 (2)	0.073 (2)	0.8503 (14)	0.077 (3)	0.669 (10)
O11A	0.3512 (13)	0.9451 (6)	0.4143 (11)	0.085 (2)	0.669 (10)
O12A	0.524 (2)	0.871 (2)	0.408 (2)	0.090 (3)	0.669 (10)
N7A	0.1472 (4)	0.4600 (5)	0.6493 (3)	0.0602 (18)	0.669 (10)
N8A	0.416 (3)	0.8789 (18)	0.4259 (18)	0.059 (4)	0.669 (10)
C40A	0.20184 (19)	0.2548 (4)	0.7124 (4)	0.0589 (9)	0.669 (10)
C41A	0.1522 (3)	0.1631 (3)	0.7650 (4)	0.0589 (9)	0.669 (10)
C42A	0.0300 (4)	0.1659 (2)	0.8035 (4)	0.0589 (9)	0.669 (10)
C43A	−0.04275 (19)	0.2603 (3)	0.7893 (4)	0.0589 (9)	0.669 (10)
C44A	0.0068 (3)	0.3520 (2)	0.7366 (4)	0.0589 (9)	0.669 (10)
C45A	0.1291 (4)	0.3493 (3)	0.6982 (4)	0.0589 (9)	0.669 (10)
C46A	0.2461 (5)	0.4821 (5)	0.6344 (3)	0.0642 (17)	0.669 (10)
C47A	0.2851 (6)	0.5852 (3)	0.5794 (2)	0.0548 (19)	0.669 (10)
C48A	0.4047 (6)	0.5938 (3)	0.5513 (3)	0.0621 (19)	0.669 (10)
C49A	0.4460 (5)	0.6894 (4)	0.5002 (5)	0.0632 (17)	0.669 (10)
C50A	0.3679 (6)	0.7764 (3)	0.4772 (6)	0.071 (3)	0.669 (10)
C51A	0.2484 (5)	0.7678 (4)	0.5053 (5)	0.075 (3)	0.669 (10)
C52A	0.2070 (5)	0.6722 (4)	0.5564 (4)	0.066 (2)	0.669 (10)
O10	−0.009 (5)	0.077 (5)	0.867 (3)	0.077 (3)	0.331 (10)
O11	0.339 (3)	0.9712 (15)	0.410 (3)	0.085 (2)	0.331 (10)
O12	0.510 (5)	0.872 (5)	0.396 (5)	0.090 (3)	0.331 (10)
N7	0.2033 (10)	0.4293 (8)	0.6474 (7)	0.063 (4)	0.331 (10)
N8	0.404 (6)	0.862 (4)	0.436 (4)	0.059 (4)	0.331 (10)
C40	0.1942 (4)	0.2784 (7)	0.7093 (8)	0.0628 (19)	0.331 (10)
C41	0.1664 (6)	0.1777 (5)	0.7574 (9)	0.0628 (19)	0.331 (10)
C42	0.0488 (7)	0.1596 (4)	0.7991 (9)	0.0628 (19)	0.331 (10)
C43	−0.0410 (4)	0.2423 (7)	0.7926 (9)	0.0628 (19)	0.331 (10)
C44	−0.0132 (6)	0.3431 (5)	0.7445 (9)	0.0628 (19)	0.331 (10)
C45	0.1044 (8)	0.3612 (4)	0.7029 (8)	0.0628 (19)	0.331 (10)
C46	0.1522 (7)	0.5239 (11)	0.6291 (6)	0.054 (3)	0.331 (10)
C47	0.2226 (11)	0.6094 (7)	0.5811 (5)	0.054 (4)	0.331 (10)
C48	0.3447 (11)	0.5931 (4)	0.5595 (6)	0.062 (4)	0.331 (10)
C49	0.4103 (7)	0.6765 (6)	0.5103 (8)	0.064 (4)	0.331 (10)
C50	0.3539 (7)	0.7762 (5)	0.4827 (9)	0.034 (3)	0.331 (10)
C51	0.2319 (7)	0.7926 (6)	0.5043 (8)	0.049 (3)	0.331 (10)
C52	0.1662 (7)	0.7092 (9)	0.5535 (6)	0.066 (4)	0.331 (10)
H1	0.55590	−0.04590	0.12530	0.0740*	
HO1	0.44750	−0.40100	0.31640	0.1250*	
H2	0.62010	−0.21740	0.18990	0.0810*	
H4	0.28340	−0.27760	0.30630	0.0770*	
H5	0.22190	−0.11010	0.23710	0.0750*	
H7	0.22210	0.00750	0.10530	0.0720*	
H9	0.10040	0.12350	0.01830	0.0770*	
H10	0.03810	0.28650	−0.06050	0.0780*	
H12	0.30080	0.40290	−0.04430	0.0850*	
H13	0.35720	0.24150	0.03850	0.0840*	
HO4	0.58390	0.02360	0.34280	0.1330*	
H14	0.61460	0.40460	0.22960	0.0770*	
H15	0.68310	0.24370	0.30790	0.0790*	
H17	0.46490	0.10560	0.25770	0.0820*	
H18	0.39870	0.26600	0.17940	0.0800*	
H20	0.33560	0.42470	0.13870	0.1080*	
H22	0.45140	0.66760	0.08890	0.0920*	
H23	0.38710	0.83310	0.00830	0.0780*	
H25	0.16580	0.69650	−0.04140	0.0830*	
H26	0.22240	0.53820	0.04390	0.0970*	
HO7	−0.06020	−0.39870	0.81320	0.1280*	
H27	0.10170	−0.11560	0.73310	0.0730*	
H28	0.05430	−0.28330	0.80120	0.0800*	
H30	−0.20170	−0.21750	0.68880	0.0790*	
H31	−0.15790	−0.04680	0.62380	0.0710*	
H33	0.17150	0.00280	0.59590	0.0710*	
H35	0.32130	0.11850	0.49910	0.0730*	
H36	0.38010	0.28180	0.42290	0.0710*	
H38	0.03760	0.40790	0.48660	0.0820*	
H39	−0.01880	0.24400	0.56500	0.0780*	
H1A0	0.04250	0.02610	0.85250	0.1150*	0.669 (10)
H40A	0.28370	0.25300	0.68660	0.0710*	0.669 (10)
H41A	0.20090	0.10000	0.77450	0.0710*	0.669 (10)
H43A	−0.12460	0.26210	0.81500	0.0710*	0.669 (10)
H44A	−0.04180	0.41520	0.72710	0.0710*	0.669 (10)
H46A	0.29820	0.43130	0.65890	0.0770*	0.669 (10)
H48A	0.45690	0.53560	0.56660	0.0750*	0.669 (10)
H49A	0.52600	0.69520	0.48140	0.0760*	0.669 (10)
H51A	0.19610	0.82600	0.48990	0.0900*	0.669 (10)
H52A	0.12700	0.66640	0.57520	0.0790*	0.669 (10)
H10A	0.03870	0.02400	0.87210	0.1150*	0.331 (10)
H40	0.27290	0.29050	0.68140	0.0750*	0.331 (10)
H41	0.22650	0.12230	0.76170	0.0750*	0.331 (10)
H43	−0.11970	0.23030	0.82050	0.0750*	0.331 (10)
H44	−0.07330	0.39850	0.74020	0.0750*	0.331 (10)
H46	0.07020	0.53720	0.64660	0.0650*	0.331 (10)
H48	0.38240	0.52640	0.57800	0.0750*	0.331 (10)
H49	0.49190	0.66560	0.49580	0.0770*	0.331 (10)
H51	0.19420	0.85930	0.48580	0.0580*	0.331 (10)
H52	0.08460	0.72010	0.56800	0.0800*	0.331 (10)

### Atomic displacement parameters (Å^2^)

**Table:**

	*U*^11^	*U*^22^	*U*^33^	*U*^12^	*U*^13^	*U*^23^
O1	0.0828 (14)	0.0652 (13)	0.0985 (16)	−0.0102 (10)	−0.0416 (12)	0.0020 (11)
O2	0.1063 (18)	0.0661 (15)	0.179 (3)	−0.0303 (13)	−0.0768 (19)	0.0141 (16)
O3	0.0831 (15)	0.0768 (15)	0.130 (2)	−0.0091 (11)	−0.0632 (14)	−0.0027 (13)
N1	0.0649 (13)	0.0607 (14)	0.0675 (14)	−0.0099 (10)	−0.0244 (11)	−0.0082 (11)
N2	0.0740 (16)	0.0640 (16)	0.0835 (17)	−0.0113 (12)	−0.0361 (13)	−0.0114 (13)
C1	0.0549 (15)	0.0656 (17)	0.0673 (16)	−0.0179 (12)	−0.0255 (12)	−0.0038 (13)
C2	0.0544 (15)	0.081 (2)	0.0685 (17)	−0.0084 (13)	−0.0273 (13)	−0.0117 (15)
C3	0.0712 (17)	0.0586 (16)	0.0600 (15)	−0.0072 (13)	−0.0309 (13)	−0.0105 (13)
C4	0.0634 (16)	0.0664 (18)	0.0587 (15)	−0.0152 (13)	−0.0205 (13)	−0.0024 (13)
C5	0.0525 (14)	0.0716 (18)	0.0614 (16)	−0.0138 (12)	−0.0143 (12)	−0.0139 (14)
C6	0.0627 (15)	0.0600 (16)	0.0555 (14)	−0.0134 (12)	−0.0220 (12)	−0.0096 (12)
C7	0.0608 (15)	0.0606 (16)	0.0584 (15)	−0.0107 (12)	−0.0214 (12)	−0.0085 (13)
C8	0.0547 (14)	0.0623 (16)	0.0562 (15)	−0.0104 (12)	−0.0162 (11)	−0.0142 (12)
C9	0.0604 (15)	0.0621 (17)	0.0747 (18)	−0.0203 (12)	−0.0270 (13)	−0.0085 (14)
C10	0.0570 (15)	0.0708 (19)	0.0754 (18)	−0.0112 (13)	−0.0295 (13)	−0.0162 (15)
C11	0.0574 (15)	0.0561 (16)	0.0613 (15)	−0.0074 (11)	−0.0213 (12)	−0.0128 (12)
C12	0.0655 (16)	0.0620 (18)	0.100 (2)	−0.0155 (13)	−0.0395 (16)	−0.0199 (16)
C13	0.0675 (17)	0.0663 (19)	0.090 (2)	−0.0060 (14)	−0.0434 (16)	−0.0184 (16)
O4	0.0802 (14)	0.0668 (13)	0.1097 (17)	−0.0073 (10)	−0.0450 (13)	0.0074 (12)
O5	0.0805 (14)	0.0945 (17)	0.1134 (18)	−0.0065 (12)	−0.0596 (14)	−0.0084 (14)
O6	0.0985 (16)	0.0586 (13)	0.1221 (19)	−0.0195 (12)	−0.0481 (14)	0.0009 (13)
N3	0.0668 (15)	0.107 (2)	0.0671 (15)	−0.0061 (14)	−0.0209 (13)	−0.0146 (15)
N4	0.0567 (13)	0.0625 (15)	0.0688 (14)	−0.0089 (11)	−0.0206 (11)	−0.0082 (12)
C14	0.0573 (15)	0.0617 (17)	0.0696 (17)	−0.0062 (12)	−0.0205 (13)	−0.0112 (13)
C15	0.0543 (15)	0.0732 (19)	0.0655 (17)	−0.0113 (13)	−0.0179 (12)	−0.0121 (14)
C16	0.0575 (15)	0.0638 (18)	0.0599 (15)	−0.0048 (13)	−0.0173 (12)	−0.0051 (13)
C17	0.0657 (16)	0.0669 (18)	0.0717 (18)	−0.0095 (13)	−0.0232 (14)	−0.0157 (15)
C18	0.0597 (15)	0.077 (2)	0.0610 (16)	−0.0077 (13)	−0.0231 (13)	−0.0099 (14)
C19	0.0610 (15)	0.0600 (17)	0.0500 (14)	0.0004 (12)	−0.0115 (12)	−0.0036 (12)
C20	0.072 (2)	0.098 (3)	0.094 (2)	−0.0238 (18)	−0.0156 (18)	−0.022 (2)
C21	0.081 (2)	0.0524 (17)	0.0654 (18)	−0.0010 (14)	−0.0042 (15)	−0.0099 (14)
C22	0.0570 (16)	0.107 (3)	0.0600 (17)	0.0058 (16)	−0.0240 (13)	−0.0185 (17)
C23	0.0583 (15)	0.0758 (19)	0.0648 (16)	−0.0127 (13)	−0.0230 (13)	−0.0163 (14)
C24	0.0530 (14)	0.0590 (16)	0.0521 (14)	−0.0025 (11)	−0.0157 (11)	−0.0128 (12)
C25	0.0657 (16)	0.0652 (19)	0.081 (2)	−0.0114 (14)	−0.0251 (15)	−0.0206 (15)
C26	0.079 (2)	0.068 (2)	0.097 (2)	−0.0099 (15)	−0.0309 (18)	−0.0186 (17)
O7	0.0836 (14)	0.0649 (13)	0.1052 (17)	−0.0191 (10)	−0.0400 (12)	0.0026 (12)
O8	0.0845 (15)	0.0623 (14)	0.148 (2)	−0.0069 (11)	−0.0510 (15)	0.0085 (14)
O9	0.0611 (12)	0.0716 (14)	0.1182 (18)	−0.0193 (10)	−0.0186 (12)	−0.0131 (12)
N5	0.0585 (13)	0.0572 (14)	0.0683 (14)	−0.0084 (10)	−0.0236 (11)	−0.0102 (11)
N6	0.0657 (15)	0.0590 (15)	0.0754 (15)	−0.0043 (11)	−0.0259 (12)	−0.0109 (12)
C27	0.0576 (15)	0.0666 (17)	0.0631 (16)	−0.0065 (12)	−0.0287 (13)	−0.0123 (13)
C28	0.0646 (16)	0.0671 (18)	0.0682 (17)	−0.0085 (13)	−0.0304 (14)	−0.0033 (14)
C29	0.0566 (15)	0.0559 (16)	0.0638 (16)	−0.0101 (12)	−0.0159 (12)	−0.0056 (13)
C30	0.0583 (15)	0.0713 (19)	0.0721 (18)	−0.0136 (13)	−0.0274 (13)	−0.0102 (15)
C31	0.0506 (13)	0.0600 (16)	0.0681 (16)	−0.0047 (11)	−0.0270 (12)	−0.0078 (13)
C32	0.0531 (14)	0.0569 (16)	0.0568 (14)	−0.0056 (11)	−0.0186 (11)	−0.0125 (12)
C33	0.0571 (15)	0.0575 (16)	0.0632 (16)	−0.0070 (12)	−0.0205 (12)	−0.0131 (13)
C34	0.0531 (14)	0.0598 (16)	0.0540 (14)	−0.0031 (11)	−0.0186 (11)	−0.0172 (12)
C35	0.0498 (13)	0.0599 (16)	0.0704 (17)	0.0000 (11)	−0.0224 (12)	−0.0158 (13)
C36	0.0471 (13)	0.0625 (17)	0.0654 (16)	−0.0031 (11)	−0.0165 (12)	−0.0171 (13)
C37	0.0589 (15)	0.0534 (15)	0.0611 (15)	−0.0105 (11)	−0.0189 (12)	−0.0152 (12)
C38	0.0516 (14)	0.0597 (17)	0.092 (2)	0.0066 (12)	−0.0254 (14)	−0.0243 (15)
C39	0.0465 (13)	0.0630 (17)	0.0822 (19)	−0.0069 (12)	−0.0166 (13)	−0.0199 (15)
O10A	0.077 (2)	0.067 (2)	0.081 (8)	−0.0226 (15)	−0.028 (5)	0.002 (5)
O11A	0.090 (4)	0.026 (5)	0.123 (2)	0.010 (4)	−0.044 (2)	0.005 (4)
O12A	0.060 (6)	0.0929 (19)	0.122 (7)	−0.022 (4)	−0.039 (3)	−0.010 (4)
N7A	0.058 (3)	0.046 (4)	0.066 (2)	−0.003 (2)	−0.025 (2)	0.005 (2)
N8A	0.063 (6)	0.046 (8)	0.066 (6)	−0.017 (4)	−0.021 (5)	−0.003 (5)
C40A	0.0567 (16)	0.0599 (16)	0.0564 (17)	−0.0131 (9)	−0.0179 (11)	−0.0057 (12)
C41A	0.0567 (16)	0.0599 (16)	0.0564 (17)	−0.0131 (9)	−0.0179 (11)	−0.0057 (12)
C42A	0.0567 (16)	0.0599 (16)	0.0564 (17)	−0.0131 (9)	−0.0179 (11)	−0.0057 (12)
C43A	0.0567 (16)	0.0599 (16)	0.0564 (17)	−0.0131 (9)	−0.0179 (11)	−0.0057 (12)
C44A	0.0567 (16)	0.0599 (16)	0.0564 (17)	−0.0131 (9)	−0.0179 (11)	−0.0057 (12)
C45A	0.0567 (16)	0.0599 (16)	0.0564 (17)	−0.0131 (9)	−0.0179 (11)	−0.0057 (12)
C46A	0.067 (3)	0.061 (3)	0.065 (3)	−0.009 (2)	−0.028 (2)	−0.007 (2)
C47A	0.042 (4)	0.065 (3)	0.060 (3)	0.004 (3)	−0.023 (3)	−0.019 (2)
C48A	0.056 (4)	0.049 (3)	0.083 (3)	−0.001 (2)	−0.037 (3)	−0.004 (2)
C49A	0.059 (3)	0.055 (3)	0.079 (3)	−0.007 (2)	−0.033 (3)	−0.008 (3)
C50A	0.074 (5)	0.095 (7)	0.053 (5)	−0.028 (5)	−0.019 (4)	−0.022 (4)
C51A	0.081 (5)	0.062 (3)	0.081 (5)	0.008 (3)	−0.039 (4)	−0.014 (3)
C52A	0.053 (3)	0.075 (5)	0.068 (3)	−0.010 (3)	−0.019 (3)	−0.013 (3)
O10	0.077 (2)	0.067 (2)	0.081 (8)	−0.0226 (15)	−0.028 (5)	0.002 (5)
O11	0.090 (4)	0.026 (5)	0.123 (2)	0.010 (4)	−0.044 (2)	0.005 (4)
O12	0.060 (6)	0.0929 (19)	0.122 (7)	−0.022 (4)	−0.039 (3)	−0.010 (4)
N7	0.059 (7)	0.056 (7)	0.082 (5)	0.011 (5)	−0.042 (5)	−0.016 (5)
N8	0.063 (6)	0.046 (8)	0.066 (6)	−0.017 (4)	−0.021 (5)	−0.003 (5)
C40	0.052 (3)	0.063 (3)	0.070 (4)	−0.0075 (17)	−0.021 (2)	−0.010 (3)
C41	0.052 (3)	0.063 (3)	0.070 (4)	−0.0075 (17)	−0.021 (2)	−0.010 (3)
C42	0.052 (3)	0.063 (3)	0.070 (4)	−0.0075 (17)	−0.021 (2)	−0.010 (3)
C43	0.052 (3)	0.063 (3)	0.070 (4)	−0.0075 (17)	−0.021 (2)	−0.010 (3)
C44	0.052 (3)	0.063 (3)	0.070 (4)	−0.0075 (17)	−0.021 (2)	−0.010 (3)
C45	0.052 (3)	0.063 (3)	0.070 (4)	−0.0075 (17)	−0.021 (2)	−0.010 (3)
C46	0.044 (4)	0.048 (6)	0.059 (5)	0.003 (4)	−0.018 (4)	−0.001 (5)
C47	0.040 (7)	0.064 (8)	0.057 (5)	−0.010 (6)	−0.012 (5)	−0.018 (5)
C48	0.028 (6)	0.066 (6)	0.094 (7)	0.017 (5)	−0.029 (6)	−0.027 (5)
C49	0.055 (6)	0.061 (8)	0.086 (8)	−0.009 (5)	−0.029 (6)	−0.022 (6)
C50	0.033 (5)	0.012 (5)	0.049 (7)	0.000 (4)	−0.014 (4)	0.004 (4)
C51	0.031 (4)	0.067 (6)	0.042 (5)	−0.011 (4)	−0.005 (4)	−0.011 (5)
C52	0.059 (6)	0.075 (9)	0.055 (5)	−0.011 (6)	−0.013 (5)	−0.007 (5)

### Geometric parameters (Å, º)

**Table:**

O1—C3	1.361 (3)	C18—H18	0.9300
O2—N2	1.210 (4)	C20—H20	0.9300
O3—N2	1.222 (4)	C22—H22	0.9300
O1—HO1	0.8100	C23—H23	0.9300
O4—C16	1.359 (4)	C25—H25	0.9300
O5—N4	1.230 (4)	C26—H26	0.9300
O6—N4	1.213 (4)	C27—C32	1.395 (4)
O4—HO4	0.8200	C27—C28	1.371 (4)
O7—C29	1.367 (4)	C28—C29	1.389 (4)
O8—N6	1.212 (4)	C29—C30	1.372 (4)
O9—N6	1.224 (4)	C30—C31	1.390 (4)
O7—HO7	0.8200	C31—C32	1.389 (4)
O10A—C42A	1.36 (3)	C33—C34	1.466 (4)
O11A—N8A	1.08 (3)	C34—C39	1.389 (4)
O12A—N8A	1.24 (5)	C34—C35	1.386 (4)
O10A—H1A0	0.8200	C35—C36	1.368 (4)
O10—C42	1.40 (6)	C36—C37	1.381 (4)
O11—N8	1.53 (7)	C37—C38	1.390 (4)
O12—N8	1.23 (10)	C38—C39	1.368 (4)
O10—H10A	0.8200	C27—H27	0.9300
N1—C6	1.414 (4)	C28—H28	0.9300
N1—C7	1.276 (4)	C30—H30	0.9300
N2—C11	1.449 (4)	C31—H31	0.9300
N3—C19	1.461 (4)	C33—H33	0.9300
N3—C20	1.187 (5)	C35—H35	0.9300
N4—C24	1.454 (4)	C36—H36	0.9300
N5—C32	1.415 (4)	C38—H38	0.9300
N5—C33	1.273 (4)	C39—H39	0.9300
N6—C37	1.450 (4)	C40A—C41A	1.390 (7)
N7A—C46A	1.240 (9)	C40A—C45A	1.391 (7)
N7A—C45A	1.483 (8)	C41A—C42A	1.389 (7)
N8A—C50A	1.50 (3)	C42A—C43A	1.390 (6)
N7—C46	1.286 (18)	C43A—C44A	1.390 (6)
N7—C45	1.520 (16)	C44A—C45A	1.390 (7)
N8—C50	1.32 (6)	C46A—C47A	1.476 (7)
C1—C6	1.383 (4)	C47A—C48A	1.391 (10)
C1—C2	1.392 (4)	C47A—C52A	1.390 (8)
C2—C3	1.376 (4)	C48A—C49A	1.390 (8)
C3—C4	1.393 (4)	C49A—C50A	1.390 (9)
C4—C5	1.367 (4)	C50A—C51A	1.390 (11)
C5—C6	1.400 (4)	C51A—C52A	1.390 (8)
C7—C8	1.460 (4)	C40A—H40A	0.9300
C8—C13	1.396 (4)	C41A—H41A	0.9300
C8—C9	1.386 (4)	C43A—H43A	0.9300
C9—C10	1.367 (4)	C44A—H44A	0.9300
C10—C11	1.384 (4)	C46A—H46A	0.9300
C11—C12	1.384 (4)	C48A—H48A	0.9300
C12—C13	1.358 (4)	C49A—H49A	0.9300
C1—H1	0.9300	C51A—H51A	0.9300
C2—H2	0.9300	C52A—H52A	0.9300
C4—H4	0.9300	C40—C45	1.390 (12)
C5—H5	0.9300	C40—C41	1.389 (13)
C7—H7	0.9300	C41—C42	1.390 (15)
C9—H9	0.9300	C42—C43	1.390 (12)
C10—H10	0.9300	C43—C44	1.390 (14)
C12—H12	0.9300	C44—C45	1.390 (15)
C13—H13	0.9300	C46—C47	1.444 (16)
C14—C15	1.382 (4)	C47—C52	1.391 (15)
C14—C19	1.384 (4)	C47—C48	1.391 (19)
C15—C16	1.383 (4)	C48—C49	1.390 (14)
C16—C17	1.392 (4)	C49—C50	1.390 (12)
C17—C18	1.371 (4)	C50—C51	1.390 (14)
C18—C19	1.390 (4)	C51—C52	1.390 (14)
C20—C21	1.505 (5)	C40—H40	0.9300
C21—C26	1.360 (5)	C41—H41	0.9300
C21—C22	1.407 (5)	C43—H43	0.9300
C22—C23	1.394 (5)	C44—H44	0.9300
C23—C24	1.383 (4)	C46—H46	0.9300
C24—C25	1.378 (4)	C48—H48	0.9300
C25—C26	1.346 (5)	C49—H49	0.9300
C14—H14	0.9300	C51—H51	0.9300
C15—H15	0.9300	C52—H52	0.9300
C17—H17	0.9300		
			
C3—O1—HO1	111.00	C27—C32—C31	118.1 (2)
C16—O4—HO4	104.00	N5—C32—C27	124.4 (3)
C29—O7—HO7	105.00	N5—C32—C31	117.5 (2)
C42A—O10A—H1A0	109.00	N5—C33—C34	122.0 (3)
C42—O10—H10A	110.00	C35—C34—C39	118.8 (2)
C6—N1—C7	118.8 (2)	C33—C34—C35	119.5 (2)
O2—N2—C11	119.3 (3)	C33—C34—C39	121.7 (2)
O3—N2—C11	118.5 (3)	C34—C35—C36	121.9 (3)
O2—N2—O3	122.2 (3)	C35—C36—C37	118.1 (3)
C19—N3—C20	119.7 (3)	C36—C37—C38	121.6 (3)
O6—N4—C24	119.3 (3)	N6—C37—C36	118.7 (2)
O5—N4—O6	122.4 (3)	N6—C37—C38	119.8 (2)
O5—N4—C24	118.3 (2)	C37—C38—C39	119.1 (3)
C32—N5—C33	118.8 (2)	C34—C39—C38	120.6 (3)
O8—N6—C37	119.4 (3)	C32—C27—H27	119.00
O9—N6—C37	118.3 (2)	C28—C27—H27	119.00
O8—N6—O9	122.3 (3)	C29—C28—H28	120.00
C45A—N7A—C46A	114.8 (6)	C27—C28—H28	120.00
O12A—N8A—C50A	113 (2)	C29—C30—H30	120.00
O11A—N8A—C50A	115 (3)	C31—C30—H30	120.00
O11A—N8A—O12A	132 (3)	C30—C31—H31	120.00
C45—N7—C46	106.0 (10)	C32—C31—H31	120.00
O11—N8—O12	105 (5)	N5—C33—H33	119.00
O11—N8—C50	126 (6)	C34—C33—H33	119.00
O12—N8—C50	129 (6)	C36—C35—H35	119.00
C2—C1—C6	121.4 (3)	C34—C35—H35	119.00
C1—C2—C3	119.3 (3)	C35—C36—H36	121.00
C2—C3—C4	120.2 (3)	C37—C36—H36	121.00
O1—C3—C4	122.4 (3)	C37—C38—H38	120.00
O1—C3—C2	117.4 (3)	C39—C38—H38	121.00
C3—C4—C5	119.8 (3)	C38—C39—H39	120.00
C4—C5—C6	121.2 (3)	C34—C39—H39	120.00
C1—C6—C5	117.9 (2)	C41A—C40A—C45A	120.0 (4)
N1—C6—C1	117.1 (2)	C40A—C41A—C42A	120.0 (4)
N1—C6—C5	125.0 (3)	O10A—C42A—C43A	122.7 (12)
N1—C7—C8	121.5 (3)	C41A—C42A—C43A	120.1 (4)
C9—C8—C13	118.4 (3)	O10A—C42A—C41A	117.1 (12)
C7—C8—C13	122.6 (3)	C42A—C43A—C44A	120.0 (4)
C7—C8—C9	119.0 (2)	C43A—C44A—C45A	120.0 (3)
C8—C9—C10	121.4 (3)	N7A—C45A—C44A	104.2 (4)
C9—C10—C11	118.4 (3)	N7A—C45A—C40A	135.9 (5)
N2—C11—C12	120.1 (2)	C40A—C45A—C44A	120.0 (4)
C10—C11—C12	121.6 (3)	N7A—C46A—C47A	123.1 (6)
N2—C11—C10	118.3 (3)	C48A—C47A—C52A	120.0 (4)
C11—C12—C13	118.7 (3)	C46A—C47A—C52A	122.4 (6)
C8—C13—C12	121.3 (3)	C46A—C47A—C48A	117.6 (5)
C6—C1—H1	119.00	C47A—C48A—C49A	120.0 (5)
C2—C1—H1	119.00	C48A—C49A—C50A	120.1 (7)
C1—C2—H2	120.00	N8A—C50A—C49A	117.9 (15)
C3—C2—H2	120.00	N8A—C50A—C51A	122.0 (15)
C3—C4—H4	120.00	C49A—C50A—C51A	120.0 (6)
C5—C4—H4	120.00	C50A—C51A—C52A	120.0 (6)
C4—C5—H5	119.00	C47A—C52A—C51A	120.0 (6)
C6—C5—H5	119.00	C45A—C40A—H40A	120.00
N1—C7—H7	119.00	C41A—C40A—H40A	120.00
C8—C7—H7	119.00	C40A—C41A—H41A	120.00
C10—C9—H9	119.00	C42A—C41A—H41A	120.00
C8—C9—H9	119.00	C44A—C43A—H43A	120.00
C9—C10—H10	121.00	C42A—C43A—H43A	120.00
C11—C10—H10	121.00	C43A—C44A—H44A	120.00
C13—C12—H12	121.00	C45A—C44A—H44A	120.00
C11—C12—H12	121.00	C47A—C46A—H46A	118.00
C8—C13—H13	119.00	N7A—C46A—H46A	118.00
C12—C13—H13	119.00	C49A—C48A—H48A	120.00
C15—C14—C19	120.6 (3)	C47A—C48A—H48A	120.00
C14—C15—C16	120.7 (3)	C50A—C49A—H49A	120.00
C15—C16—C17	119.4 (3)	C48A—C49A—H49A	120.00
O4—C16—C17	123.0 (3)	C50A—C51A—H51A	120.00
O4—C16—C15	117.6 (3)	C52A—C51A—H51A	120.00
C16—C17—C18	119.1 (3)	C51A—C52A—H52A	120.00
C17—C18—C19	122.3 (3)	C47A—C52A—H52A	120.00
C14—C19—C18	117.9 (2)	C41—C40—C45	120.0 (8)
N3—C19—C18	127.9 (3)	C40—C41—C42	120.0 (7)
N3—C19—C14	114.2 (2)	O10—C42—C41	134 (3)
N3—C20—C21	122.3 (4)	C41—C42—C43	120.0 (8)
C20—C21—C22	122.0 (3)	O10—C42—C43	105 (3)
C20—C21—C26	118.1 (3)	C42—C43—C44	120.0 (8)
C22—C21—C26	120.0 (3)	C43—C44—C45	120.0 (7)
C21—C22—C23	120.3 (3)	N7—C45—C44	154.3 (8)
C22—C23—C24	116.7 (3)	C40—C45—C44	120.0 (8)
N4—C24—C23	119.6 (2)	N7—C45—C40	85.7 (8)
C23—C24—C25	122.6 (3)	N7—C46—C47	119.8 (10)
N4—C24—C25	117.8 (2)	C46—C47—C52	118.5 (11)
C24—C25—C26	119.7 (3)	C48—C47—C52	120.0 (9)
C21—C26—C25	120.8 (3)	C46—C47—C48	121.5 (9)
C19—C14—H14	120.00	C47—C48—C49	120.0 (8)
C15—C14—H14	120.00	C48—C49—C50	120.0 (10)
C14—C15—H15	120.00	N8—C50—C49	127 (3)
C16—C15—H15	120.00	N8—C50—C51	113 (3)
C18—C17—H17	120.00	C49—C50—C51	120.1 (9)
C16—C17—H17	120.00	C50—C51—C52	120.0 (8)
C19—C18—H18	119.00	C47—C52—C51	120.0 (10)
C17—C18—H18	119.00	C41—C40—H40	120.00
C21—C20—H20	119.00	C45—C40—H40	120.00
N3—C20—H20	119.00	C40—C41—H41	120.00
C23—C22—H22	120.00	C42—C41—H41	120.00
C21—C22—H22	120.00	C42—C43—H43	120.00
C22—C23—H23	122.00	C44—C43—H43	120.00
C24—C23—H23	122.00	C43—C44—H44	120.00
C26—C25—H25	120.00	C45—C44—H44	120.00
C24—C25—H25	120.00	N7—C46—H46	120.00
C25—C26—H26	120.00	C47—C46—H46	120.00
C21—C26—H26	120.00	C47—C48—H48	120.00
C28—C27—C32	121.3 (3)	C49—C48—H48	120.00
C27—C28—C29	119.8 (3)	C48—C49—H49	120.00
C28—C29—C30	119.9 (3)	C50—C49—H49	120.00
O7—C29—C30	117.5 (3)	C50—C51—H51	120.00
O7—C29—C28	122.6 (3)	C52—C51—H51	120.00
C29—C30—C31	120.2 (3)	C47—C52—H52	120.00
C30—C31—C32	120.6 (3)	C51—C52—H52	120.00
			
C7—N1—C6—C1	149.3 (3)	C17—C18—C19—N3	179.7 (3)
C7—N1—C6—C5	−30.5 (4)	C17—C18—C19—C14	2.0 (4)
C6—N1—C7—C8	−179.1 (2)	N3—C20—C21—C26	170.0 (4)
O2—N2—C11—C10	−168.2 (3)	N3—C20—C21—C22	−9.0 (6)
O3—N2—C11—C10	13.3 (4)	C20—C21—C22—C23	179.9 (3)
O2—N2—C11—C12	12.2 (4)	C22—C21—C26—C25	1.0 (5)
O3—N2—C11—C12	−166.3 (3)	C26—C21—C22—C23	0.9 (5)
C20—N3—C19—C14	−170.7 (3)	C20—C21—C26—C25	−178.1 (3)
C20—N3—C19—C18	11.5 (5)	C21—C22—C23—C24	−1.1 (4)
C19—N3—C20—C21	−178.3 (3)	C22—C23—C24—N4	177.5 (2)
O5—N4—C24—C23	−178.2 (3)	C22—C23—C24—C25	−0.5 (4)
O5—N4—C24—C25	−0.2 (4)	N4—C24—C25—C26	−175.7 (3)
O6—N4—C24—C23	1.4 (4)	C23—C24—C25—C26	2.4 (4)
O6—N4—C24—C25	179.5 (3)	C24—C25—C26—C21	−2.6 (5)
C33—N5—C32—C31	−145.5 (3)	C32—C27—C28—C29	0.5 (4)
C32—N5—C33—C34	179.5 (2)	C28—C27—C32—N5	−178.9 (3)
C33—N5—C32—C27	35.9 (4)	C28—C27—C32—C31	2.5 (4)
O9—N6—C37—C38	166.6 (3)	C27—C28—C29—O7	178.7 (3)
O8—N6—C37—C36	171.0 (3)	C27—C28—C29—C30	−2.5 (4)
O9—N6—C37—C36	−11.6 (4)	C28—C29—C30—C31	1.4 (4)
O8—N6—C37—C38	−10.8 (4)	O7—C29—C30—C31	−179.7 (3)
C45A—N7A—C46A—C47A	174.5 (4)	C29—C30—C31—C32	1.6 (4)
C46A—N7A—C45A—C44A	159.3 (5)	C30—C31—C32—C27	−3.5 (4)
C46A—N7A—C45A—C40A	−19.8 (9)	C30—C31—C32—N5	177.7 (2)
O11A—N8A—C50A—C51A	2 (3)	N5—C33—C34—C35	−173.2 (3)
O12A—N8A—C50A—C49A	2 (3)	N5—C33—C34—C39	6.2 (4)
O11A—N8A—C50A—C49A	−175.6 (18)	C33—C34—C35—C36	−179.6 (3)
O12A—N8A—C50A—C51A	179.1 (19)	C39—C34—C35—C36	1.1 (4)
C2—C1—C6—N1	−175.4 (2)	C33—C34—C39—C38	−179.7 (3)
C2—C1—C6—C5	4.3 (4)	C35—C34—C39—C38	−0.4 (4)
C6—C1—C2—C3	−2.2 (4)	C34—C35—C36—C37	−0.3 (4)
C1—C2—C3—C4	−1.5 (4)	C35—C36—C37—C38	−1.2 (4)
C1—C2—C3—O1	179.3 (2)	C35—C36—C37—N6	177.0 (2)
C2—C3—C4—C5	2.8 (4)	C36—C37—C38—C39	1.9 (4)
O1—C3—C4—C5	−178.0 (3)	N6—C37—C38—C39	−176.3 (3)
C3—C4—C5—C6	−0.5 (4)	C37—C38—C39—C34	−1.1 (4)
C4—C5—C6—C1	−3.0 (4)	C41A—C40A—C45A—C44A	0.0 (8)
C4—C5—C6—N1	176.7 (3)	C45A—C40A—C41A—C42A	−0.1 (8)
N1—C7—C8—C13	−0.7 (4)	C41A—C40A—C45A—N7A	179.1 (6)
N1—C7—C8—C9	177.2 (3)	C40A—C41A—C42A—C43A	0.2 (8)
C9—C8—C13—C12	2.3 (4)	C40A—C41A—C42A—O10A	177.0 (11)
C7—C8—C9—C10	178.6 (3)	C41A—C42A—C43A—C44A	−0.1 (8)
C13—C8—C9—C10	−3.4 (4)	O10A—C42A—C43A—C44A	−176.7 (12)
C7—C8—C13—C12	−179.9 (3)	C42A—C43A—C44A—C45A	−0.1 (8)
C8—C9—C10—C11	1.1 (4)	C43A—C44A—C45A—N7A	−179.2 (5)
C9—C10—C11—N2	−177.1 (3)	C43A—C44A—C45A—C40A	0.1 (8)
C9—C10—C11—C12	2.4 (4)	N7A—C46A—C47A—C48A	−167.3 (5)
N2—C11—C12—C13	176.0 (3)	N7A—C46A—C47A—C52A	13.4 (7)
C10—C11—C12—C13	−3.6 (4)	C46A—C47A—C48A—C49A	−179.3 (5)
C11—C12—C13—C8	1.2 (4)	C52A—C47A—C48A—C49A	0.0 (7)
C19—C14—C15—C16	0.3 (4)	C46A—C47A—C52A—C51A	179.3 (5)
C15—C14—C19—N3	−179.8 (3)	C48A—C47A—C52A—C51A	0.0 (7)
C15—C14—C19—C18	−1.7 (4)	C47A—C48A—C49A—C50A	0.0 (9)
C14—C15—C16—C17	0.9 (4)	C48A—C49A—C50A—N8A	177.2 (12)
C14—C15—C16—O4	−179.2 (3)	C48A—C49A—C50A—C51A	0.0 (11)
C15—C16—C17—C18	−0.7 (4)	N8A—C50A—C51A—C52A	−177.1 (13)
O4—C16—C17—C18	179.4 (3)	C49A—C50A—C51A—C52A	0.0 (11)
C16—C17—C18—C19	−0.7 (4)	C50A—C51A—C52A—C47A	0.0 (9)

### Hydrogen-bond geometry (Å, º)

Cg2, Cg3, Cg4 and Cg8 are the centroids of the C8–C13, C14–C19, C21–C26 and C47*A*–C52*A* benzene rings, respectively.

**Table:**

*D*—H···*A*	*D*—H	H···*A*	*D*···*A*	*D*—H···*A*
O1—H*O*1···O9^i^	0.81	2.05	2.836 (3)	163
O4—H*O*4···O12*A*^i^	0.82	2.16	2.89 (3)	148
O7—H*O*7···O3^ii^	0.82	2.06	2.832 (4)	158
C1—H1···O6^iii^	0.93	2.53	3.417 (4)	159
C14—H14···O2^iii^	0.93	2.59	3.289 (4)	132
C49*A*—H49*A*···O12*A*	0.93	2.30	2.63 (3)	100
C2—H2···*Cg*2^iv^	0.93	3.00	3.610 (3)	125
C15—H15···*Cg*8^v^	0.93	2.99	3.665 (4)	131
C36—H36···*Cg*3	0.93	2.81	3.487 (3)	130
C43*A*—H43*A*···*Cg*4^vi^	0.93	2.80	3.539 (5)	137
C43—H43···*Cg*4^vi^	0.93	2.94	3.590 (11)	128

Symmetry codes: (i) *x*, *y*−1, *z*; (ii) *x*, *y*−1, *z*+1; (iii) −*x*+1, −*y*+1, −*z*; (iv) −*x*+1, −*y*, −*z*; (v) −*x*+1, −*y*+1, −*z*+1; (vi) −*x*, −*y*+1, −*z*+1.
