# Supplementary figures and images for: Crystal structure of 4-[(E)-(4-nitro­benzyl­idene)amino]­phenol
Source: Acta Crystallogr E Crystallogr Commun. 2015 Jan 17;71(Pt 2):o113–4. doi: 10.1107/S2056989015000511 (PMC4384620; doi:10.1107/S2056989015000511)

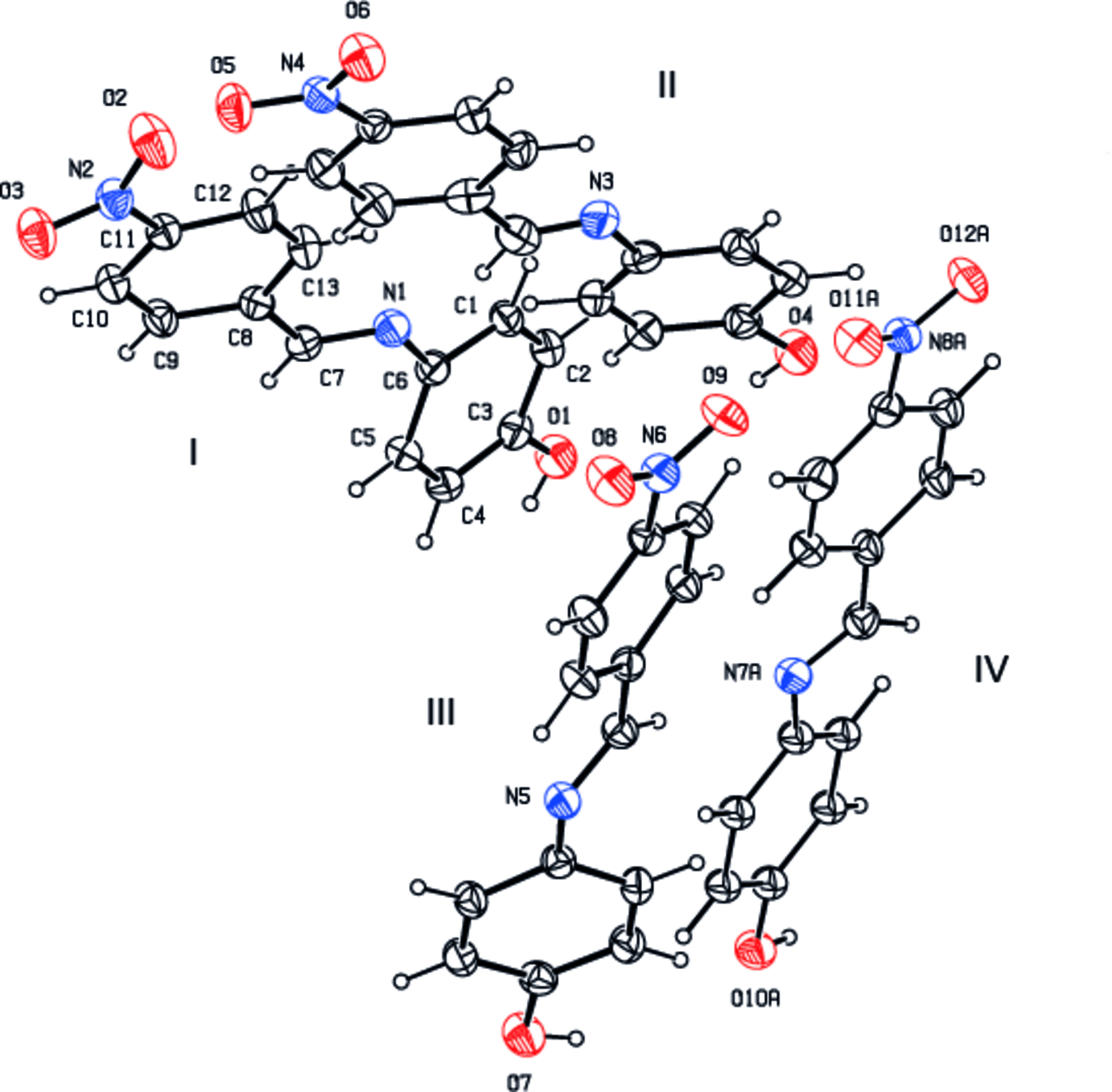

Supplement: Supplementary file 4 [file e-71-0o113-fig1.tif]

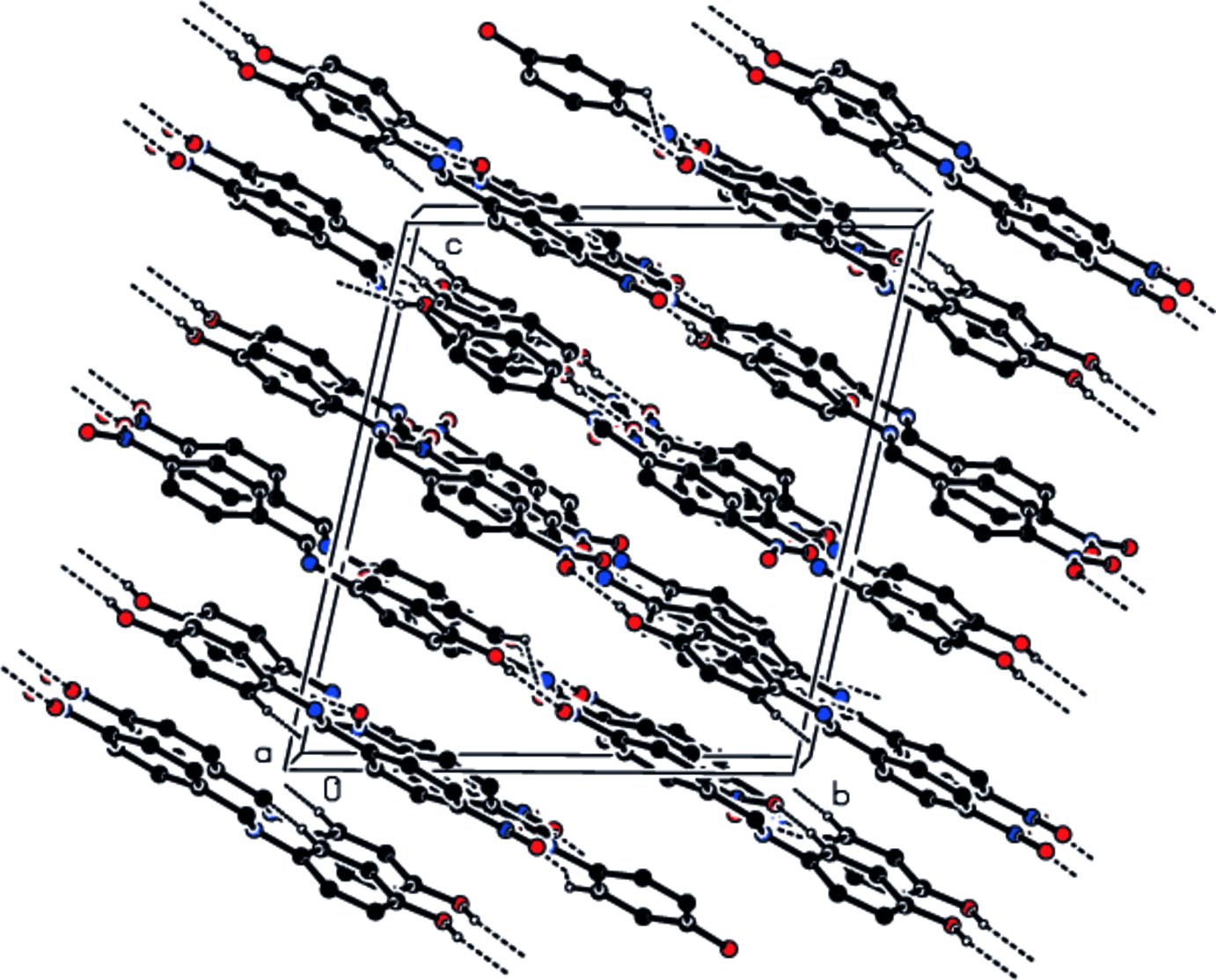

Supplement: Supplementary file 5 [file e-71-0o113-fig2.tif]
